# Supplementary material for: m6A Methylases Regulate Myoblast Proliferation, Apoptosis and Differentiation
Source: Animals (Basel). 2022 Mar 18;12(6):773. doi: 10.3390/ani12060773 (PMC8944832; doi:10.3390/ani12060773)
Supplement: Supplementary file 1 [file animals-12-00773-s001.zip › Supplementary figure and legends.pdf]

Supplementary Figure

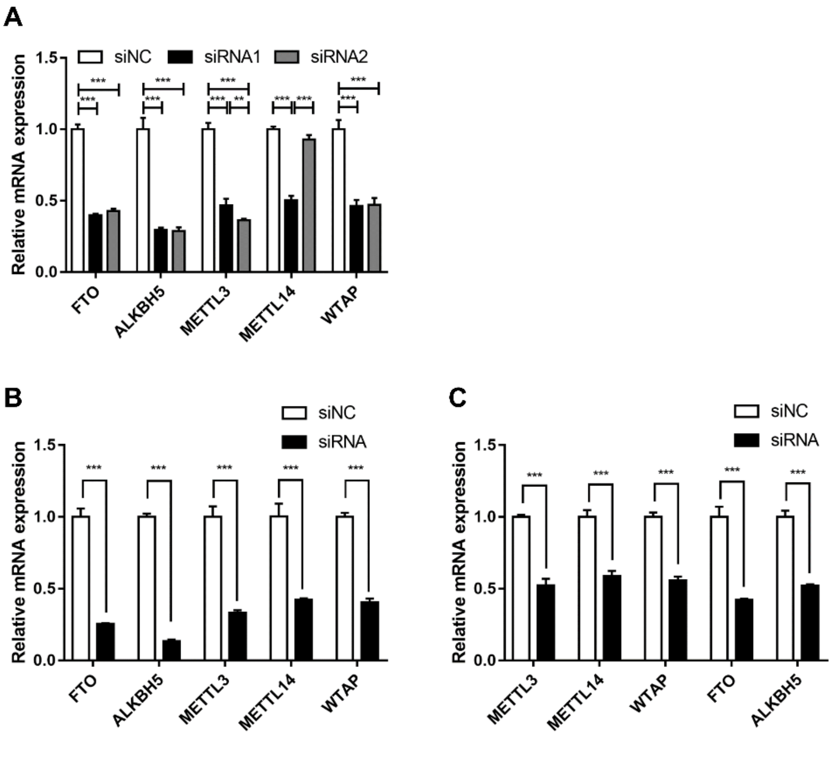

Figure S1. Interference efficiency of siRNAs in myoblast proliferation and differentiation. (A) The relative mRNA expression of FTO, ALKBH5, METTL3, METTL14 and WTAP were measured by RT-qPCR in myoblasts transfected with siRNAs for 48 h, respectively. The siFTO-1, siALKBH5-2, siMETTL3-2, siMETTL14-1 and siWTAP-1 with the highest interference efficiency were selected for subsequent experiments. (B) The relative mRNA expression of FTO, ALKBH5, METTL3, METTL14 and WTAP in myoblasts transfected with the siRNAs for 36 h of cell growth. (C) The relative mRNA expression of FTO, ALKBH5, METTL3, METTL14 and WTAP on day 3 of myogenic differentiation in myoblasts transfected with siRNAs. Results are presented as the means ± SD from three independent experiments. *$p < 0.05$, **$p < 0.01$, ***$p < 0.001$, using Student's t test.
