# Supplementary material for: m6A Methylases Regulate Myoblast Proliferation, Apoptosis and Differentiation
Source: Animals (Basel). 2022 Mar 18;12(6):773. doi: 10.3390/ani12060773 (PMC8944832; doi:10.3390/ani12060773)
Supplement: Supplementary file 1 [file animals-12-00773-s001.zip › Table S1. Primers used in this study.pdf]

Table S1. Primers used in this study.

Primer name	Sequence (5′-3′)
Primers for RT-qPCR	
qGAPDH-F	AGTTCAACGGCACAGTCAAGG
qGAPDH-R	ACCACATACTCAGCACCAGCA
qMYOD1-F	AACCCCAACCCGATTTACC
qMYOD1-R	CACAACAGTTCCTTCGCCTCT
qMYOG-F	GGCGTGTAAGGTGTGTAAG
qMYOG-R	CTTCTTGAGTCTGCGCTTCT
qMYF6-F	GTGATAACTGCCAAGGAAGGAG
qMYF6-R	CGAGGAAATGCTGTCCACGA
qMYH3-F	TGAACGCCCTCTCCAAATCC
qMYH3-R	AATGAAGTGCTGTCTCGGCA
qMYMK-F	TCGGCCATCCTCATCATTG
qMYMK-R	CGTACGTGTAGTCCCAGTCCTC
qCKM-F	CAACATGAAGGAGGTTTTCCG
qCKM-R	GGTTAGATGGGCAGGTGAGC
qMETTL3-F	TCGAAAGCTGCACTTCAGAC
qMETTL3-R	TCCAACGCTCTGTGTAAGGG
qMETTL14-F	TGACATCAGAGAACTGACACCC
qMETTL14-R	AGGTCCAATCCTTCCCCAGA
qWTAP-F	GCCTGGAAGTTTACGCCTGA
qWTAP-R	TCCTGACTGCTTTTAAGCTCCT
qFTO-F	AGCAGCGTACAACGTCACTT
qFTO-R	AGGGTCGTCCTCACTTTCCT
qALKBH5-F	TACTTCTTCGGCGAGGGCTA
qALKBH5-R	TGGTAGTCGTTGATGACGGC
qCCNA2-F	GCAGCCTTTCATTTAGCACTCT
qCCNA2-R	ATTGACTGTTGTGCGTGCTG
qCCNB1-F	TACCCATTCACCATTATCAA
qCCNB1-R	ACTAACTATGCTGGACTACGA
qCCNE1-F	CGATGTCTCTGTTCGCTCCA
qCCNE1-R	CCACACTGGCTTCTCACAGT
qCDK1-F	AGTGGAAACCAGGAAGCTTAG
qCDK1-R	ATTCGTTTGGCAGGATCATAGA
qPCNA-F	CCTTGGTGCAGCTAACCCTT
qPCNA-R	TTGGACATGCTGGTGAGGTT
qMCM6-F	TCTTCATGGAGGATTACAGTGCG
qMCM6-R	CGAGATTGACATCAGGTGTTTCC
qBCL-XL-F	CACTGTGCGTGGAAAGCGTA
qBCL-XL-R	GCTGCATTGTTCCCGTAGAG
qBCL2-F	ATGTGTGTGGAGAGCGTCAA
qBCL2-R	ATACAGCTCCACAAAGGCGT
qBAD-F	TCCCAGAGTTTGAGCAGAGTG
qBAD-R	TTAGCCAGTGCTTGCTGAGAC
qBAX-F	ATCGGAGATGAATTGGACAGTAACAT
qBAX-R	TGAGCACTCCAGCCACAAAGA
qCASP3-F	GTGGTGCTGAGGATGACA
qCASP3-R	CACAAAGAGCCTGGATGAA
qCASP6-F	ATCGGGAAACTGTGAATGG

qCASP6-R	ATTGAGGCAAAGCAGGGA
Primers for plasmid construction	
METTL3-cds-F	ctagctagcATGTCGGACACGTGGAGC
METTL3-cds-R	cggggtaccCTATAGATTCTTAGGTTTAGAG
METTL14-cds-F	ctagctagcATGGACAGCCGCTTGCAG
METTL14-cds-R	cggggtaccCTATCGAGGTGGAAAGCC
WTAP-cds-F	ctagctagcATGACCAACGAAGAACCTC
WTAP-cds-R	cggggtaccTTACAAAACTGAACCCTGT
FTO-cds-F	ctagctagcATGAAGCGGACCCCGACG
FTO-cds-R	ccgctcgagCTAGGGCCTGGTTTCCAG
ALKBH5-cds-F	ctagctagcATGGCGGCCGCCAGCGGCTA
ALKBH5-cds-R	cggggtaccTTAGTGCCGCCGCATCTTCA
