# Supplementary material for: m6A Methylases Regulate Myoblast Proliferation, Apoptosis and Differentiation
Source: Animals (Basel). 2022 Mar 18;12(6):773. doi: 10.3390/ani12060773 (PMC8944832; doi:10.3390/ani12060773)
Supplement: Supplementary file 1 [file animals-12-00773-s001.zip › Table S2. siRNAs used in this study.pdf]

Table S2. siRNAs used in this study.

siRNA name	Sequence (5′-3′)
siFTO-F1	CCAUAAAGAGGUUCAACAATT
siFTO-R1	UUGUUGAACCUCUUUAUGGTT
siFTO-F2	GCUGUGCUUCGCGAAGUUATT
siFTO-R2	UAACUUCGCGAAGCACAGCTT
siALKBH5-F1	GCUUCAGCUCCGAGAACUATT
siALKBH5-R1	UAGUUCUCGGAGCUGAAGCTT
siALKBH5-F2	GGAUACGCUGCUGAUGAAATT
siALKBH5-R2	UUUCAUCAGCAGCGUAUCCTT
siMETTL3-F1	GCACUUGGAUCUUCGGAAUTT
siMETTL3-R1	AUUCCGAAGAUCCAAGUGCTT
siMETTL3-F2	CCACAUGGAUACCUGCAAATT
siMETTL3-R2	UUUGCAGGUAUCCAUGUGGTT
siMETTL14-F1	GCCGUCUUCCAGAGAACAATT
siMETTL14-R1	UUGUUCUCUGGAAGACGGCTT
siMETTL14-F2	CCUCCUCCCAAAUCUAAAUTT
siMETTL14-R2	AUUUAGAUUUGGGAGGAGGTT
siWTAP-F1	GCAAGUACACAGAUCUCAATT
siWTAP-R1	UUGAGAUCUGUGUACUUGCTT
siWTAP-F2	CCAGGGACGUAUUGCACAATT
siWTAP-R2	UUGUGCAAUACGUCCCUGGTT
