# Supplementary material for: m6A Methylases Regulate Myoblast Proliferation, Apoptosis and Differentiation
Source: Animals (Basel). 2022 Mar 18;12(6):773. doi: 10.3390/ani12060773 (PMC8944832; doi:10.3390/ani12060773)
Supplement: Supplementary file 1 [file animals-12-00773-s001.zip › Table S3. Antibodies used in this study.pdf]

Table S3. Antibodies used in this study.

Antibodies	Catalog and Source
anti-PAX7	ab187339, Abcam
anti-MyoD1	ab16148, Abcam
anti-METTL3	ab195352, Abcam
anti-METTL14	26158-1-AP, Proteintech
anti-WTAP	60188-1-Ig, Proteintech
anti-FTO	ab126605, Abcam
anti-ALKBH5	ab195377, Abcam
anti-GAPDH	ab181603, Abcam
anti-CCNB1	55004-1-AP, Proteintech
anti-CCND2	10934-1-AP, Proteintech
anti-CCNE1	MBS820219, Mybinsource
anti-CDK1	ab133327, Abcam
anti-PCNA	ab92552, Abcam
anti-p21	YT3497, Immunoway
anti-BCL-XL	ab32370, Abcam
anti-BCL2	D160117, Sangon Biotech
anti-BAD	ab90435, Abcam
anti-BAX	D120073, Sangon Biotech
anti-CASP3	19677-1-AP, Proteintech
anti-MYH3	sc-53091, Santa Cruz
anti-MYOG	ab264517, Abcam
anti-ACTN1	sc-17829, Santa Cruz
HRP-conjugated goat anti-mouse IgG	D110087, Sangon Biotech
HRP-conjugated goat anti-rabbit IgG	D110058, Sangon Biotech
Alexa Fluor 555-conjugated donkey anti-rabbit IgG	ab150074, Abcam
Alexa Fluor 488-conjugated goat anti-mouse IgG	ab150113, Abcam
Alexa Fluor 555-conjugated donkey anti-mouse IgG	ab150106, Abcam
